# Evaluation of Active LDPE Films for Packaging of Fresh Orange Juice

**DOI:** 10.3390/polym15010050

**Published:** 2022-12-23

**Authors:** Pedro V. Rodrigues, Dalila M. Vieira, Paola Chaves Martins, Vilásia Guimarães Martins, M. Cidália R. Castro, Ana V. Machado

**Affiliations:** 1 Instituto de Polímeros e Compósitos, Universidade do Minho, Campus de Azurém, 4800-058 Guimarães, Portugal; 2 Laboratory of Food Technology, School of Chemistry and Food, Federal University of Rio Grande (FURG), Rio Grande 96203-900, Brazil

**Keywords:** active packaging, orange juice, physicochemical characteristics, microbial stability, shelf-life

## Abstract

Microbial development, enzymatic action, and chemical reactions influence the quality of untreated natural orange juice, compromising its organoleptic characteristics and causing nutritional value loss. Active low-density polyethylene (LDPE) films containing green tea extract (GTE) were previously prepared by a blown film extrusion process. Small bags were prepared from the produced films, which were then filled with fresh orange juice and stored at 4 °C. Ascorbic acid (AA) content, sugar content, browning index, color parameters, pH, total acidity (TA) and microbial stability were evaluated after 3, 7, and 14 days of storage. The packaging containing GTE maintained the microbial load of fresh juice beneath the limit of microbial shelf-life (6 log CFU/mL) for the bacterial growth, with a more prominent effect for LDPE with 3%GTE. Regarding yeasts and molds, only the CO_LDPE_3GTE package maintained the microbial load of fresh juice below the limit for up to 14 days. At 14 days, the lowest levels of AA degradation (32.60 mg/100 mL of juice) and development of brown pigments (browning index = 0.139) were observed for the packages containing 3% of GTE, which had a pH of 3.87 and sugar content of 11.4 g/100 mL of juice at this time. Therefore, active LDPE films containing 3% of GTE increase the shelf-life of fresh juice and can be a promising option for storage of this food product while increasing sustainability.

## 1. Introduction

Fruit juices are known for being a source of vitamins, soluble/insoluble fiber and minerals, and their characteristic flavor makes them a product of high consumption [1]. In fact, processed products, specifically juice, are very popular because they are easily consumed [2]. The processing and storage conditions, packaging and raw material are very important factors for the stability of citrus juice as these factors determine the microbiological, enzymatic, chemical, and physical changes that can spoil the juice’s sensory and nutritional characteristics [1,3,4]. 

The high content of vitamin C (ascorbic acid), an essential nutrient for humans, in orange juice and its pleasant taste makes it the most appreciated and consumed citrus juice [1,2,5,6,7]. However, due to its nature, vitamin C can oxidize and be lost during the juice storage period. Its degradation rate is highly dependent on storage conditions such as temperature, dissolved oxygen, and the oxygen permeability of the packaging material [2,8].

Fresh orange juice is extremely susceptible to microbial growth, which results in fast deterioration. The deterioration of the organoleptic and physicochemical characteristics is a major reason for the rejection of juice by customers [9]. Lactic and acetic acid bacteria have been isolated from fruit juices, and many microorganisms found in juices are acid-tolerant bacteria and fungi [10,11]. Usually, the most reported bacteria genera include *Acetobacter, Alicyclobacillus, Bacillus, Gluconobacter, Lactobacillus, Leuconostoc, Zymomonas*, and *Zymobacter* [12,13].

The availability of nutrients, presence of antimicrobial compounds, oxidation-reduction potential, water activity, and pH are the critical factors that influence the spoilage of juices, with the last two factors being of crucial importance. The spoilage in juices results in degradation of the product, which induces changes in appearance, color, texture, CO_2_ production, cloud loss and the development of off-flavors [3,11,14,15]. The acidic properties of fruit juices (pH < 4.5) act as a vital barrier to microbial growth. However, foodborne pathogens such as, *E. coli* and *Salmonella* can persist at an acidic pH level due to the acid stress response. Thus, in the last twenty years, several foodborne outbreaks related to unpasteurized fruit juices have been reported in several countries [11,13,16,17]. 

The shelf-life of a food product is commonly recognized as the length of time during which it is still suitable for consumption and sale with acceptable characteristics under specified storage conditions, and is determined by its sensory characteristics (color, aroma and taste). On a food label, shelf-life can be indicated by either a “best before” date that indicates the quality of the food or a “use by” date that is linked to food safety. The accuracy of the shelf-life prediction displayed on the package is important for both food industries and consumers [18]. Active compounds can provide several functions when incorporated into the packaging materials, which are an alternative to conventional packaging systems [19,20]. There are several natural antioxidants, sweeteners, coloring and antimicrobial agents originating from animals, plants, or even microorganisms, although they have not been defined as a specific category for natural additives [21]. Several natural substances can have an active function in the package. These include essential oils (EOs) or extracts of plants that are known as Generally Recognized as Safe (GRAS). They can be used as food additives not only to extend the shelf-life but also to preserve the food’s quality for a long period of time [22,23,24,25,26,27,28]. The EOs/extracts have the potential function of inhibiting microorganisms and reducing lipid oxidation due to their high content in phenolic compounds and volatile terpenoids [27,28,29]. These compounds have several biological properties, such as antioxidant and antimicrobial activities [26,27,28,30,31,32,33,34,35,36,37,38,39,40]. Through disruption of the cytoplasmic membrane, the active components of plant EOs/extracts inhibit microorganism proliferation. In fact, these components disrupt the electron flow, active transport and proton motive forces and inhibit protein synthesis [28,29]. The use of EOs/extracts in food could decrease or substitute the dependence on synthetic antioxidants and antimicrobial compounds, thereby meeting the consumer’s demand for more natural products [26,27].

Since consumers demand more natural products, much research has been conducted aiming to replace synthetic compounds with natural ones, such as plant extracts and EOs, due to their benefits to human health. GTE is rich in flavonols and gallic acid derivatives, namely, (+)-catechin, (-)-epicatechin, (+)-gallocatechin, (-)-epicatechin gallate, (-)-epigallocatechin, and (-)-epigallocatechin gallate. GTE is described as a powerful source of polyphenol antioxidants and already has the status of food additive [20,39,40,41,42,43]. Studies on GTE have revealed its excellent antioxidant properties and nontoxicity in various food model systems, which encourages its incorporation into polymer matrixes for the development of active packaging films to prevent food oxidation [42,43,44,45,46].

This active packaging could help the food packaging industry to eliminate or reduce spoilage and foodborne pathogens on the surface of products, and thereby, increase a product’s shelf life. Thus, active packaging incorporating antimicrobial activity is one the most promising methods to extend shelf-life while sustaining the nutritional and sensory quality of food [47,48]. Moreover, in recent years, researchers have made efforts to develop alternatives to multi-material packages in order to improve their recyclability. Thus, LDPE packaging systems have been produced with the incorporation of nanoparticles or natural compounds to increase juice preservation [7,49,50,51]. Therefore, the purpose of this study is to evaluate the potential of GTE in a LDPE matrix as an innovative packaging to preserve and extend orange juice shelf-life.

## 2. Materials and Methods

### 2.1. Materials

Low-density polyethylene (LDPE) was kindly provided by Vizelpas—Flexible Films, S.A. (Portugal) and GTE was supplied from ESSÊNCIAD’UMSEGREDO, LDA (Portugal). Distilled water, phenolphthalein (indicator ACS, Merck, Darmstadt, Germany), and sodium hydroxide (NaOH ≥ 98%, Merck, Darmstadt, Germany) were used in total acidity (TA) tests. Ethanol (absolute ≥ 99%, Thermo Fisher Scientific, Waltham, MA, USA) was used in the browning index assay. Sulfuric acid (H_2_SO_4_ ≥ 99%, Merck, Darmstadt, Germany), starch, iodine solution (Merck, Darmstadt, Germany), and sodium thiosulfate (Na_2_S_2_O_3_ ≥ 99.99%, Merck, Darmstadt, Germany) were used for the determination of ascorbic acid (AA) content. Peptone water, plate count agar (PCA), and Dichloran Rose-Bengal Chloramphenicol agar (DRBC) were acquired from Merck (Germany) and were used in microbiological tests. 

### 2.2. Preparation of LDPE Active Films

LDPE active films were prepared according to the methodology already reported by our group [46]. Briefly, a co-rotating twin-screw extruder (Leistritz AG LSM 34 6L) was used to prepare a masterbatch of LDPE/10 wt.% GTE, at 170 °C. Then, this was diluted into neat LDPE to produce monolayer and coextruded films of LDPE containing 1.5 and 3 wt.% of GTE by blown film extrusion.

Descriptions of the different active films produced for juice packaging based on LDPE containing GTE are shown in Table 1. 

### 2.3. Packaging Orange Juice

To evaluate the packaging potential, an orange juice was selected which was produced under ideal hygienic-sanitary conditions and without the addition of any preservatives or preservation processes. Before the production of juice, the oranges were sanitized with water and soap and all the equipment used in the process was previously sterilized in an autoclave (Technal, AV-18, São Paulo, Brazil). The oranges were peeled and the juice was extracted using a juicer. The juice produced containing pieces of orange pulp was standardized with a 1 mm mesh filter and stored in a sterile glass container. The films used for application were previously decontaminated using a laminar flow cabinet with UV light (15 W) (Solab, SLH-656/4, New York, NY, USA) for 15 min of exposure on each side. Then, small bags (14 × 13 cm) made from the produced films were sealed at the bottom, filled with 300 mL of juice and closed aseptically.

### 2.4. Storage

The orange juice packages were stored in the dark and in cold conditions (4 °C). The samples from a single package for each treatment were assessed with a total of 5 tests for physicochemical properties including color, pH, sugar content, TA, browning index, AA content and microbiological growth. The tests were conducted immediately after packaging and after 3, 7, and 14 storage days. 

### 2.5. Measurement of Color, pH and Sugar Content

The juices were evaluated for color variations using a colorimeter (Minolta Chroma Meter, CR-400, Konica Minolta, NJ, USA) with triplicate measurements. The equipment uses the CIELab measurement system that measures the L* parameter (lightness index scale) in a range from 0 (black) to 100 (white), the a* parameter that indicates the degree of red (+a) or green (−a*) color and the b* parameter which measures the degree of yellow (+b) or blue (−b*) color.

The pH measurement was performed using a pH meter (Even, PHS-3E, USA) at room temperature. The sugar concentration was measured using a refractometer (Hanna, HI 96801, Judetul Cluj, Romania) which provides values referring to the amount in mg of sugar in 100 mL of juice.

### 2.6. Total Acidity (TA)

To determine the TA, 5 mL of previously filtered juice was used, and homogenized with 25 mL of distilled water with 2 drops of 1% phenolphthalein solution. The mixture was titrated with 0.1 M NaOH until a pink color appeared. TA was calculated using Equation (1) [52]:

(1) $$\mathrm{TA}\;=\;\frac{\left(V\;\times \;M\;\times \;100\right)}{p}$$

where V (mL) is the volume of NaOH spent in the titration of the juice; M is the molarity of the standardized NaOH solution, and p (mL) is the amount of juice used.

### 2.7. Browning Index Measurement

A 10 mL sample of juice was collected from the package and centrifuged at 2000 rpm for 20 min. The supernatant was homogenized in a 1:1 ratio with ethanol and filtered with a 0.45-mm filter paper to obtain a clarified extract. The extract absorbance was read on a UV-Vis. spectrophotometer at 420 nm [53].

### 2.8. Determination of Ascorbic Acid (AA)

The AA content was calculated according to Zambiazi [52], where 20 ml of juice was mixed with 3 mL of H_2_SO_4_ (12 M) and 3 mL of starch (0.5% *m*/*v*). After homogenization, the mixture was titrated with a standardized 0.01 M iodine solution until a dark color appeared. Afterward, the solution was titrated again using 0.01 M sodium thiosulfate until the dark color disappeared; finally, the solution was titrated one more time with 0.01 M iodine until the reappearance of the dark color. The amount of AA present was calculated by applying Equation (2):

(2) $$\mathrm{AA}\;=\; [\left(\mathrm{Vi}\;\times \;\mathrm{Fi}\right)\;-\;\left(\mathrm{Vt}\;\times \;\mathrm{Ft}\right)]\;\times \;0.88$$

where AA is the content of ascorbic acid present in the juice expressed in mg of ascorbic acid/mL of juice, Vi (mL) is the total volume of iodine used in the titrations, Fi is the correction factor obtained in the standardization of the iodine solution, Vt (mL) is the volume of sodium thiosulfate used in the titration, and Ft is the correction factor for the standardized sodium thiosulfate solution.

### 2.9. Microbiological Growth Tests

The microbiological growth of the bacteria, molds and yeasts in the juice were evaluated. A sample of 1 mL of juice was aseptically collected for each different treatment and diluted in tubes containing 9 mL of 0.1% (*w*/*v*; peptone/water) sterile peptone water. The tubes containing juice and peptone water were manually shaken for approximately 1 min at room temperature (25 °C). Then, the serial dilutions of the homogenates were prepared for each treatment (10^0^ to 10^−6^). For the growth of bacteria, 1 mL of each dilution was added to petri plates and placed on plate count agar for in-depth homogenization. The plates were incubated at 37 °C for 48 h, and subsequently counted for the determination of colony forming units (CFU). For the determination of the molds and yeasts, 100 μL of each dilution was taken and inoculated on the surface of dicloran rose bengal agar. The plates were incubated in an oven incubator at 25 °C for 5 days, and after that, the colonies were counted as described above. All the microbiological tests were performed in triplicate [54].

### 2.10. Statistical Analysis

The resulting data was evaluated with Microsoft Windows Excel 365 and OriginPro (Version 17) software. At least three replicates were used to express the results as mean ± standard deviation. For the color analysis, an analysis of variance (ANOVA) was applied as well as the Tukey’s test to determine significant differences with a 95% significance interval. The software used was Statistics 5.0.

## 3. Results and Discussion

### 3.1. Ascorbic Acid (AA) and Browning Index

**Figure 1:**
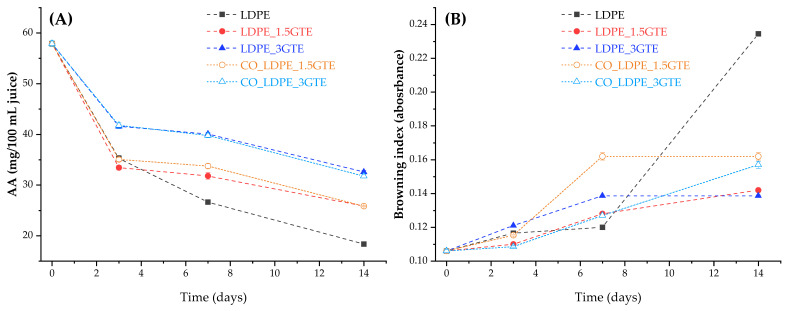
The content of AA (**A**), and browning index (**B**) in orange juice packed with neat LDPE films and active LDPE films containing GTE stored at 4 °C for 14 days.

The evolution of AA content in orange juice packed in active LDPE and in LDPE film, stored at 4 °C for 14 days, is shown in Figure 1A. The film that presented the best retention of AA was LDPE_3GTE followed by CO_LDPE_3GTE, while the LDPE without GTE had poor retention of AA, as expected. Since oxygen is one of the main components that contribute to AA degradation and considering that the headspace was the same for all packages, the only factor that can explain these variations in AA retention is oxygen permeability [2]. In fact, the results indicate that the LDPE_3GTE film had the lowest permeability followed by CO_LDPE_3GTE, LDPE_1.5GTE, and CO_LDPE_1.5GTE film, respectively. Considering the limit of 20 mg/100 mL of AA value for shelf-life estimation [55], all LDPE active films presented a higher value than the AA limit after storage for 14 days, whereas the lowest value, 25.83 mg/100 mL, was obtained for LDPE_1.5GTE and CO_LDPE_1.5GTE film. During the early stage of storage, the results indicate a fast degradation of AA, which was followed by a gradual loss. This agrees with results obtained by other researchers [56,57], and can be attributed mostly to the oxygen dissolved in the juice and in the headspace of the package at the beginning of storage [53]. Indeed, the dissolved oxygen concentration has a great impact on the AA oxidation rate. Solomon et al. [58] and Wilson et al. [59] demonstrate that the rate of oxidation of AA is significantly associated with the level of dissolved oxygen and with the duration of storage time. Throughout the storage period, the oxygen permeation across the packaging contributes significantly to the extension of the aerobic mechanism of AA oxidation in the active LDPE films [53,60]. Parameters such as light, heat, oxygen, enzymes, and peroxides stimulated the oxidative process of AA [7,8].

Overall, after 14 days of storage, the final AA content in the juice varied from 18.36 to 32.60 mg/100 mL. Comparing these data with the minimum values recommended for processed orange juice, it can be seen that they were lower than the value indicated as minimum for industrialized juice, 40 mg/100 mL [61].

During the storage of citrus products, non-enzymatic processes are one of the most critical chemical phenomena responsible for quality and color variations. Moreover, ascorbic acid degradation into dehydroascorbic acid (DHA) is known as the main chemical reaction that occurs during the storage of all kinds of juice. More specifically, the resulting DHA is converted to 2,3-diketogulonic acid (DKG), forming xylosone through the aerobic pathway, which degrades to form reductones or ethylglyoxal. Then, these compounds react with amino acids, yielding brown compounds. Therefore, there is a high-level of correlation between the percentage loss of ascorbic acid and the increase in the browning index [6,53,62]. This relationship is noticeable in Figure 1, where a significant decrease in the AA content is observed for the packages during the storage at 4 °C, while browning index, only increased slightly. The values of the browning index in fresh juice measured immediately after packaging were 0.106 (Figure 1B). Leizerson and Shimoni [63] reported values of the browning index up to 0.367, which leads to the conclusion that is still undetected in our case. It can also be seen in Figure 1B that the package with only LDPE is the one that exhibits the highest browning index, as expected, following by a pronounced increase from day 7 onwards. The package that demonstrated the lowest browning index was LDPE_3GTE, which proves the influence of GTE as an antioxidant agent. In fact, Roig et al. [57] and Bharate et al. found a relationship between the browning index and the oxidative loss of L-ascorbic acid in citrus juices [62]. The results obtained for the browning index are similar to the Zerdin et al. [53] study that determined the extent of AA loss due to oxygen and temperature for orange juice packed in oxygen scavenging film and oxygen barrier film. For the browning index, Cortés et al. [64], obtained lower values (0.093) than those obtained in the present study; however, the temperature used in their study (2 °C) was lower. According to the results of Emamifar et al. [7], the browning index increased significantly for all packaging tested stored at 4 °C, agreeing with the values reached in this study.

### 3.2. Color

The color of an orange juice is a crucial characteristic for the consumer’s initial purchasing decision and for consumer perception about the food quality. Carotenoid pigments are responsible for orange juice color and can be affected by product ripening, processing treatments, storage conditions and browning reactions [7,8]. Table 2 depicts the evolution of color parameters of orange juice packed in active LDPE films with and without GTE, stored at 4 °C. It can be noticed that color parameters did not show significant variations until 3 days of storage. After this time, L* values start to increase, suggesting an increase in the brightness and light; a* and b* parameters did not present significant variations until 14 days of storage. Thus, there was no considerable variation in juice color. The L* parameter increased in LDPE_1.5GTE after 7 days but at a lower rate than for CO_LDPE_1.5GTE. Moreover, it was observed that LDPE_3GTE and CO_LDPE_1.5GTE were the samples with higher brightness after 14 days. Concerning the a* parameter (variation between red and green color), an increase in a* value was verified with a higher amount of GTE (LDPE_3GTE =−0.13 ± 0.03) when compared with only LDPE (−1.26 ± 0.05), after 14 days storage. Parameter b* (variation between yellow and blue color) showed an increase for all packaging after 14 days of storage, with the largest increase for packaging with 3% GTE. Thus, a color shift toward positive b* and negative a* directions indicate greater values of yellow and green colors in the orange juice. These changes show the progressive deterioration of the juice due to changes in the color spectrum.

These color changes have a good correlation with the reduction of AA content and the development of brown pigments during storage. A bleaching effect may be due to the oxidation of carotenoids; consequently, the free radicals formed might be responsible for the changes in the orange juice color [7,8]. Bull et al. reported an increase in the total color variation with time during storage in fresh orange juice, regardless of treatment [65]. Esteve et al. obtained a slight decrease in L* at 4 °C for different commercial orange juices [61]. Lee and Coates studied pasteurized orange juice and reported a small increase in L* value from 40.22 to 41.22 [66]. Rivas et al. describe a decrease in the parameter L* for pasteurized orange-carrot juice during refrigerated storage, and Cortés et al. observed that L* values increased substantially after one week of refrigerated storage, which is also in agreement with the findings of this study [64,67].

### 3.3. pH and Total Acidity (TA)

**Figure 2:**
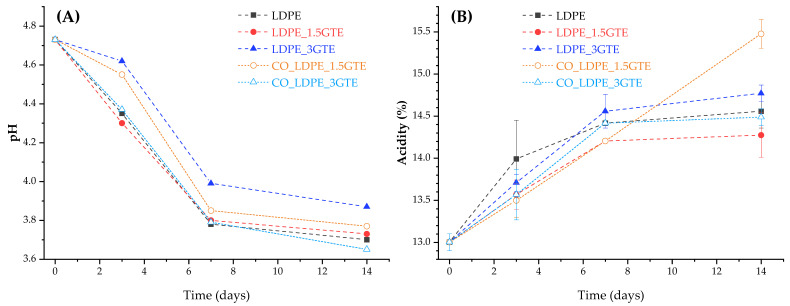
pH indexes (**A**), and TA (**B**) of orange juice packed in active LDPE films containing GTE and stored at 4 °C for 14 days.

After 14 days of storage at 4 °C, the pH values of the juice studied in five different packages were within the normal range (3–4), however, with significant differences among them, as presented in Figure 2A. After 7 days of storage at 4 °C, the pH values of the juice in different packages decrease from 4.73 (initial) to close to 3.85 (day 7), where the CO_LDPE_3GTE and LDPE films had a larger decrease in pH values. In fact, the results obtained are in agreement with the study by Touati et al. [68], which found that pH values become significantly lower with storage, independent of the temperature. In addition, Bull et al. observed a significant variation in pH in studies of pasteurized and high pressurized orange juices stored for 12 weeks [65]. In contrast, Esteve et al. [61] did not detected significant changes in pH values of various pasteurized orange and carrot-orange juices refrigerated at 4 °C and 10 °C, and in the study by Cortés et al. [64], there was a statistically significant increase in pH values for all the juices analyzed. This increase can be related to a microbiological deterioration of juice, as described by Del Caro et al. [69], who studied the changes in pH in citrus segments and juices during storage at 4 °C.

When fermentation of orange juice occurs, the organic acids (produced from the biochemical process due to the development of spoilage microorganisms) lead to pH reductions which results in a specific flavor and palatability of the juice. In general, acid environments protect against the growth of pathogens [9]. Citric acid is the most abundant free acid in orange juice, followed by malic acid, and although in limited quantities, they also appear as citrates or malates giving a buffer effect to orange juice. Non-volatile free acids, such as oxalic, galacturonic and quinic acids and many others are found in smaller quantities [61]. As would be expected, higher acidity corresponds to lower pH value. Owing to the presence of this natural buffer medium in orange juice (based on mainly potassium citrates and malates), pH variations are slightly more pronounced than acidity variations. The TA in the five studied packages present similar behavior (Figure 2B), except for the package made of CO_LDPE_1.5GTE film. During storage, acidity increased in all juices until day 7, reaching a plateau. However, the CO_LDPE_1.5GTE film exhibited a linear increase in total acidity with time, indicating the start of spoilage or fermentation of the sample. These results are in agreement with those reported by Esteve et al. [58] and by Supraditareporn and Pinthong [70] where a significant increase in acidity with storage time was observed. The low pH values of orange juices (3–4) significantly limit the number and types of bacteria that can survive or grow, especially the lactic acid bacteria, which are spoilage microorganisms that cause the development of slime, gas, off-flavors, turbidity, and changes in acidity [70]. 

### 3.4. Microbiological Analysis and Sugar Content

**Figure 3:**
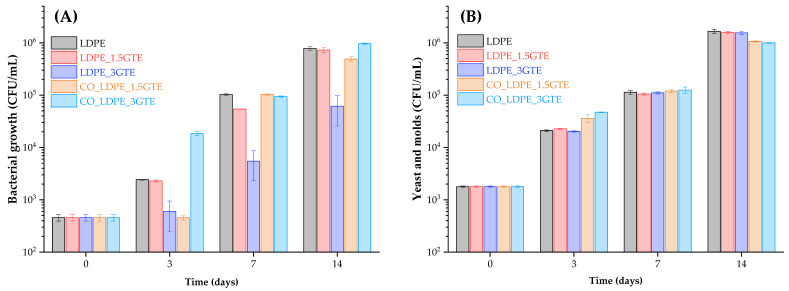
Count of bacterial (**A**) and yeast and molds (**B**) growth in orange juice packed from active LDPE films containing GTE stored at 4 °C for 14 days.

At the moment of packaging, the initial population of microorganisms inside the orange juice was 1.79 × 10^3^ CFU/mL for yeast and molds and 4.57 × 10^2^ CFU/mL for bacteria (Figure 3). These results indicate that despite the large population of bacteria, the yeast and molds increased during storage, meaning that yeast and molds are better adapted than bacteria to orange juice under refrigeration, as reported by Sadler et al. [71] and Emamifar et al. [7]. Figure 3 shows that the population of yeast and molds, and bacterial growth increased to 1.55 × 10^6^ CFU/mL and 6.15 × 10^4^ CFU/mL, respectively, after 14 days of storage inside the LDPE_3GTE package. Nevertheless, a significant deceleration was observed in the growth rate and in the total count of bacteria population after 7 days of storage, especially for LDPE_3GTE films. 

For fresh orange juice, the shelf-life is defined as the required time need to reach a microbial population of 6 log CFU/mL [72]. Moreover, previous studies have shown a shelf life of 14 days for refrigerated orange juice (4 °C) [5,65,73,74]. The average population of bacterial growth remained below 6 log CFU/mL until 7 days in all the packages, yet in the case of yeast and molds, only the CO_LDPE_3GTE package remained below 6 log films at 7 days. It is noteworthy that as the GTE concentration increases, the antimicrobial activity is enhanced, yet for the same GTE concentrations, the LDPE_3GTE package exhibited a higher antibacterial activity compared with CO_LDPE_3GTE, even after 14 days of storage. Considering the results obtained, it is possible to verify that the increased GTE concentration in the packages has a more prominent effect on antibacterial activity than on antifungal activity after a week of storage, and the LDPE_3GTE packages maintained the same pattern over time, always having higher antimicrobial activity than the other active packages. Thus, yeast, molds, and bacteria exhibit different levels of sensitivity to the GTE incorporated in active LDPE films. Published studies demonstrate that the yeast growth during storage is the principal parameter that affects the shelf life of natural orange juice [1,74]. 

Muriel-Galet et al. characterized the antimicrobial efficiency of polypropylene/ethylene-vinyl alcohol (EVOH) films with oregano essential oil and citral and verified that antimicrobial activity reduced spoilage flora on salad and was more effective against Gram-negative bacteria [75]. Another study assessed the antimicrobial effect of GTE and oregano essential oil incorporated in EVOH films, which showed strong antimicrobial activity against the tested microorganisms, and films containing GTE also inhibited the growth of *L. monocytogenes* and *E. coli* in liquid media; however, a synergistic antimicrobial effect was not detected [39]. The study of Dong et al. [28] based on bilayer LDPE active packaging with the incorporation of rosemary and cinnamon essential oils revealed an effective retardation of the growth of the total viable count in Pacific white shrimps, showing that the cinnamon essential oil exhibited stronger antimicrobial effects than rosemary essential oil.

**Figure 4:**
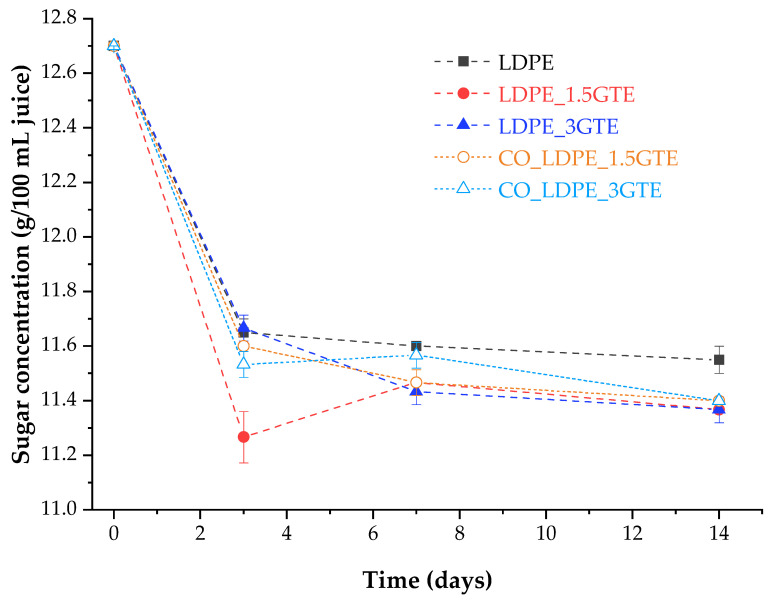
Sugar content in orange juice packed in active LDPE films stored at 4 °C for 14 days.

The initial value of sugar concentration in orange juice for the different packages was 12.7 g/100 mL. After 3 days of storage, orange juice showed a decrease in sugar concentration by about 9.45% in all packages, where juice in the LDPE_1.5GTE packaging film showed the higher reduction (≈11%). From the day 3 to the end of storage (14 days), the sugar concentration remained practically constant, as can be seen in Figure 4. The reduction in sugars concentration is correlated with the increase in microorganism growth in the juice, as can be seen in Figure 3 and Figure 4. As yeast and mold populations increase, there is a consumption of sugars that are transformed into carbon dioxide through a fermentation process and, which consequently, contributes to a decrease in the sugar concentrations.

## 4. Conclusions

The results show that final content of AA after 14 days of storage varied from 18.36 to 32.60 mg/100 mL, which is lower than the reference value for industrialized juice (40 mg/100 mL). The decrease in the amount of AA is correlated with an increase in browning values; thus, the LDPE packages had the greatest decrease in AA content and also the highest browning index. The juices with higher AA content and lowest browning index were the ones packed in film containing 3% GTE (both monolayer and co-extruded). Other parameters, such as pH and TA, showed different patterns. Although pH decreased by approximately 21%, the TA increased. The most promising packaging for increasing the juice shelf life was verified through microbiological analysis. This analysis showed that the increased GTE concentration in the LDPE films had a more pronounced effect on the bacteria than the fungi after a week of storage. Therefore, it can be concluded that GTE is more effective as an inhibitor of bacterial growth in orange juice.

It is important to mention that the orange juice used in this study is a natural juice without any additional preservatives and usually has a short shelf life between 3 and 4 days. Overall, the microbiological activity of the produced active LDPE films demonstrates that, at least 14 days are necessary for the growth of bacteria, yeasts and molds to reach the limit value of 6 log. The addition of GTE had a positive effect on the inhibition of bacterial growth, being most effective for the monolayer film with 3% GTE.

Based on the results from this investigation, we conclude that the LDPE_3GTE package is the most suitable for storage of orange juice for 14 days at 4 °C. Juice stored in this package maintained a higher concentration of AA, had a lower browning index and had the most resistance to bacterial growth. Thus, active LDPE films containing GTE are effective as a new approach to preserve and extend the shelf-life of fresh orange juice at 4 °C.

As a general conclusion, to achieve their desired properties as a gas/light barrier or for mechanical stability, conventional food packaging systems are made of multi-material products. For example, Tetra Pak^@^ packages have paperboard, aluminum, and LDPE layers, which have a complex manufacturing process and are difficult to recycle. Therefore, a package made of a single polymer will have lower production costs, a smaller carbon footprint as well as increased shelf-life and sustainability.

## Figures and Tables

**Table 1:** Packaging films based on LDPE/GTE.

Film	Description
LDPE	Monolayer film of LDPE
LDPE_1.5GTE	Monolayer film of LDPE with 1.5 wt.% GTE
LDPE_3GTE	Monolayer film of LDPE with 3 wt.% GTE
CO_LDPE_1.5GTE	LDPE film coextruded with LDPE_1.5GTE
CO_LDPE_3GTE	LDPE film coextruded with LDPE_3GTE

**Table 2:** The color parameters obtained for orange juice packaged in active LDPE films containing GTE at different days of storage.

Orange Juice in Film:		LDPE	LDPE 1.5GTE	LDPE 3GTE	CO_LDPE 1.5GTE	CO_LDPE 3GTE
Day 0	L*	41.47 ^a^	40.95 ^a^	39.04 ^b^	40.96 ^a^	40.29 ^a,b^
	a*	−1.67 ^d^	−1.10 ^c^	0.30 ^a^	−1.25 ^c^	−0.87 ^b^
	b*	20.10 ^a^	20.04 ^a^	18.32 ^b^	18.80 ^b^	18.78 ^b^
Day 3	L*	40.67 ^c^	41.59 ^b,c^	42.98 ^a^	42.94 ^a^	42.57 ^a,b^
	a*	−0.50 ^b,c^	−0.37 ^b^	0.87 ^a^	−1.25 ^d^	−0.77 ^c^
	b*	20.17 ^c^	22.77 ^b^	25.96 ^a^	22.89 ^b^	23.42 ^b^
Day 7	L*	43.05 ^b^	42.56 ^b^	46.15 ^a^	47.10 ^a^	43.67 ^b^
	a*	−0.64 ^c^	−0.91 ^d^	0.79 ^a^	−0.09 ^b^	−0.94 ^d^
	b*	23.36 ^b,c^	22.46 ^c^	29.07 ^a^	28.79 ^a^	24.26 ^b^
Day 14	L*	46.65 ^a^	43.33 ^c^	45.65 ^a,b^	45.51 ^b^	43.41 ^c^
	a*	−1.26 ^c^	−0.59 ^b^	−0.13 ^a^	−1.10 ^c^	−0.96 ^c^
	b*	28.03 ^a^	24.27 ^c,d^	26.40 ^b^	25.40 ^b,c^	23.68 ^d^

Different superscript letters in the same line indicate a statistically significant difference (*p* < 0.05). Values are given as mean, and standard deviation values are under 5% for all samples.

## Data Availability

The data presented in this study is available in the article.
